# Clinical impact of pneumomediastinum in patients with myositis-associated interstitial lung disease

**DOI:** 10.1371/journal.pone.0328043

**Published:** 2025-07-11

**Authors:** Junghee Jung, Min Jee Kim, Bin Yoo, Chang-Keun Lee, Yong-Gil Kim, Seokchan Hong, Soo Min Ahn, Ho Cheol Kim

**Affiliations:** 1 Department of Pulmonary and Critical Care Medicine, Asan Medical Center, University of Ulsan College of Medicine, Seoul, Republic of Korea; 2 Division of Rheumatology, Department of Internal Medicine, Asan Medical Center, University of Ulsan College of Medicine, Seoul, Republic of Korea; University of Texas McGowan Medical School at Houston, UNITED STATES OF AMERICA

## Abstract

**Background:**

Idiopathic inflammatory myositis (IIM) frequently coexists with interstitial lung disease (ILD), significantly impacting morbidity and mortality. Spontaneous pneumomediastinum, a complication of myositis-associated ILD, remains understudied regarding its clinical implications.

**Method:**

We retrospectively reviewed patients diagnosed with myositis-associated ILD at Asan Medical Center, Seoul, South Korea, from April 2012 to September 2023. Patients were categorized into two groups based on the presence or absence of spontaneous pneumomediastinum during the follow-up period.

**Results:**

Among the 70 patients included in the study, the median age was 55.9 ± 12.2 years, with 62.9% being female. Pneumomediastinum developed in 12 (17.1%) patients. Clinical characteristics did not significantly differ between the pneumomediastinum and non-pneumomediastinum groups, except for the subtype of IIM. Notably, pneumomediastinum was observed in 11 (91.7%) patients with dermatomyositis and 1 (8.3%) with anti-synthetase syndrome (ASS), but none with polymyositis. Multivariate analysis revealed pneumomediastinum as a significant risk factor for mortality (hazard ratio: 2.829, 95% confidence interval: 1.100–7.270, *p* = 0.031) after adjusting for other variables. Patients with pneumomediastinum exhibited worse survival compared with patients without pneumomediastinum (median survival time: 77.7 ± 11.7 vs. 13.6 ± 3.7 months, *p* = 0.013).

**Conclusion:**

Spontaneous pneumomediastinum is an independent risk factor for mortality in patients with myositis-associated ILD.

## Introduction

Idiopathic inflammatory myositis (IIM) constitutes a group of autoimmune disorders characterized by inflammation and weakness of skeletal muscles, further categorized into subtypes such as dermatomyositis (DM), polymyositis (PM), inclusion body myositis (IBM), anti-synthetase syndrome (ASS), and overlap myositis [1]. Although proximal skeletal muscles are most commonly affected, manifestations can extend to peripheral myositis and systemic extramuscular involvement [2].

Among the extramuscular presentations of IIM, pulmonary invasion is frequently observed and often manifests as interstitial lung disease (ILD) [3–5]. In some cases, pulmonary involvement may manifest as the initial and sole indication of IIM [5]. Myositis-associated ILD is notorious for its high morbidity and mortality rates, especially among patients with DM and PM [6]. Of particular concern is spontaneous pneumomediastinum, a rare yet potentially fatal complication of myositis-associated ILD [7,8]. Despite its significance, studies examining spontaneous pneumomediastinum in patients with myositis-associated ILD are limited [9,10]. Therefore, this study aims to elucidate the clinical implications of pneumomediastinum on overall outcomes in patients with myositis-associated ILD.

## Methods

### Study population

This retrospective analysis was conducted at a single center, utilizing data collected from patients of Asan Medical Center who were diagnosed with myositis-associated ILD between April 2012 and September 2023. Diagnosis of IIM was confirmed by a rheumatologist following the European League Against Rheumatism/American College of Rheumatology (EULAR/ACR) classification criteria for adult and juvenile idiopathic inflammatory myopathies [11], or by documentation of International Classification of Diseases, 10th Revision (ICD-10) codes M33.1, M33.2, or M33.9, along with registration in the national Rare and Intractable Disease (RID) support program. The study included three subclasses of IIM: DM, PM, and ASS. Diagnosis of myositis-associated ILD was established by an attending pulmonologist based on compatible findings from chest computed tomography (CT) at the time of diagnosis. Among the 75 identified patients, those meeting the following criteria were excluded: (i) inability to be subclassed into DM, PM, or ASS (n = 3), (ii) pre-existing diagnosis of connective tissue disease (CTD)-associated ILD before the onset of myositis-associated ILD (n = 1), (iii) loss to follow- during the study period (n = 1).

### Clinical data

Laboratory data, including aldolase, lactate dehydrogenase (LD), and Krebs von den Lungen-6 (KL-6) levels, were obtained from blood tests conducted closest time to the diagnosis of myositis-associated ILD. The occurrence of spontaneous pneumomediastinum was assessed using chest X-rays or CT scans obtained between the diagnosis of myositis-associated ILD and the final follow-up, including death.

Patients were categorized into two groups; the pneumomediastinum group and the non-pneumomediastinum group. Events such as death or bilateral lung transplantation during the study period were considered as “death”. Clinical and survival data were retrieved from the National Health Insurance of Korea database, encompassing regular outpatient care and hospitalizations. Spirometry results, including forced vital capacity (FVC), total lung capacity (TLC), and diffusing capacity of the lung for carbon monoxide (DLco), expressed percentages of the expected normal values, were analyzed. Additionally, results from the 6-minute walk test (6MWT) were analyzed in accordance with the guidelines from the American Thoracic Society/European Respiratory Society [12,13]. This study was conducted in compliance with the principles outlined in the Declaration of Helsinki. The Institutional Review Board (IRB) of Asan Medical Center approved the study protocol (IRB no. 2023−1394), and waived the requirement for informed consent given the use of anonymized clinical data and the retrospective nature of the study design. Data were accessed in December 2023. Authors had access to information that could identify individual participants during and after data collection according to IRB protocols.

### Statistical analysis

Continuous variables were presented as mean ± standard deviation. Categorial variables were analyzed using χ^2^ or Fisher’s exact test, while differences in continuous variables were assessed using Student’s *t*-test. Continuous variables that did not adhere to normal distribution were compared using the Mann–Whitney U test.

Logistic regression and Cox proportional hazard models were utilized to identify risk factors associated with pneumomediastinum and mortality, respectively. Variables with a p-value of less than 0.2 were included in multivariate analysis. Survival analyses were conducted using the Kaplan–Meier. All P-values were two-tailed, with statistical significance defined as *p* < 0.05. Statistical analyses were performed using SPSS 23.0.

## Results

### Patients

A total of 70 patients were ultimately included in the analysis, with 12 (17.1%) developing pneumomediastinum during follow-up. The median age of the cohort was 55.9 ± 12.2 years, with 62.9% (44/70) being female. A total of 90% (n = 63/70) patients underwent muscle biopsy. Most baseline characteristics, as detailed in Table 1, did not exhibit significant differences between the pneumomediastinum and non-pneumomediastinum groups, except for the subtype of IIM. There was a significant difference in the distribution of IIM subtypes between the pneumomediastinum and non-pneumomediastinum groups (*p* = 0.024). Dermatomyositis was more prevalent in individuals with spontaneous pneumomediastinum (11/12 (91.7%) compared with those who did not 33/58 (56.9%)), whereas no cases of polymyositis were observed in the pneumomediastinum group.

**Table 1:** Comparison of baseline characteristics between the pneumomediastinum and non-pneumomediastinum groups.

Characteristics	Total (*n* = 70)	Pneumomediastinum (*n* = 12)	Non-Pneumomediastinum (*n* = 58)	*p-*value
Age, years	55.9 ± 12.2	54.1 ± 10.1	56.3 ± 12.6	0.572
Ever-smoker	21 (30%)	2 (16.7%)	19 (32.8%)	0.325
Female	44 (62.9%)	9 (75.0%)	35 (60.3%)	0.514
Combined malignancy	7 (10%)	0 (0.0%)	7 (12.1%)	0.343
Baseline PFT				
FVC, % predicted (n = 62)	59.6 ± 15.4	59.7 ± 13.7	59.6 ± 15.7	0.988
FEV_1_, % predicted (n = 62)	63.1 ± 13.5	62.6 ± 14.7	63.2 ± 13.4	0.892
DL_CO_, % predicted (n = 57)	45.5 ± 16.0	35.3 ± 9.0	46.6 ± 16.3	0.103
TLC, % predicted (n = 53)	68.3 ± 14.7	63.8 ± 8.5	68.8 ± 15.2	0.480
6MWT (n = 50)				
Meter, m	387.0 ± 113.4	382.6 ± 66.6	387.4 ± 117.9	0.929
Lowest SpO_2_ ≤ 88%	11 (22%)	1 (20%)	10 (22.2%)	>0.999
Initial baseline laboratory data				
Aldolase, IU/L (n = 67)	15.2 ± 17.4	8.7 ± 3.7	16.7 ± 18.9	0.151
LD, U/L (n = 69)	447.5 ± 207.1	517.5 ± 194.5	432.8 ± 208.3	0.200
KL-6 > 1000.0U/ML (n = 56)	24 (42.9%)	6 (66.7%)	18 (8.3%)	0.151
Chest CT data				
Traction bronchiectasis	31 (43.3%)	8 (66.7%)	23 (39.7%)	0.086
Honeycombing	1 (1.4%)	0	1 (1.7%)	0.647
Cyst	5 (7.1%)	1 (8.3%)	4 (6.9%)	> 0.999
Emphysema	6 (8.6%)	0	6 (10.3%)	0.581
ILD visual extent over 20%	30 (42.9%)	7 (58.3%)	23 (39.7%)	0.234
IIM subtypes				0.024
Dermatomyositis	44 (62.9%)	11 (91.7%)	33 (56.9%)	
Polymyositis	21 (30.0%)	0 (0.0%)	21 (36.2%)	
Anti-synthetase syndrome	5 (7.1%)	1 (8.3%)	4 (6.9%)	

Data are presented as mean ± standard deviation, frequencies (proportions), or median (interquartile range).

PFT = pulmonary function test; FVC = forced vital capacity; FEV_1_ = forced expiratory volume in 1 second; DL_CO_ = diffusing capacity of the lung for carbon monoxide; TLC = total lung capacity; 6MWT = 6-minute walk test; SpO_2_ = arterial oxygen saturation; LD = lactose dehydrogenase; KL-6 = Krebs von den Lugen-6; CT = computed tomography; ILD = interstitial lung disease; IIM = idiopathic inflammatory myositis.

**Fig 1:**
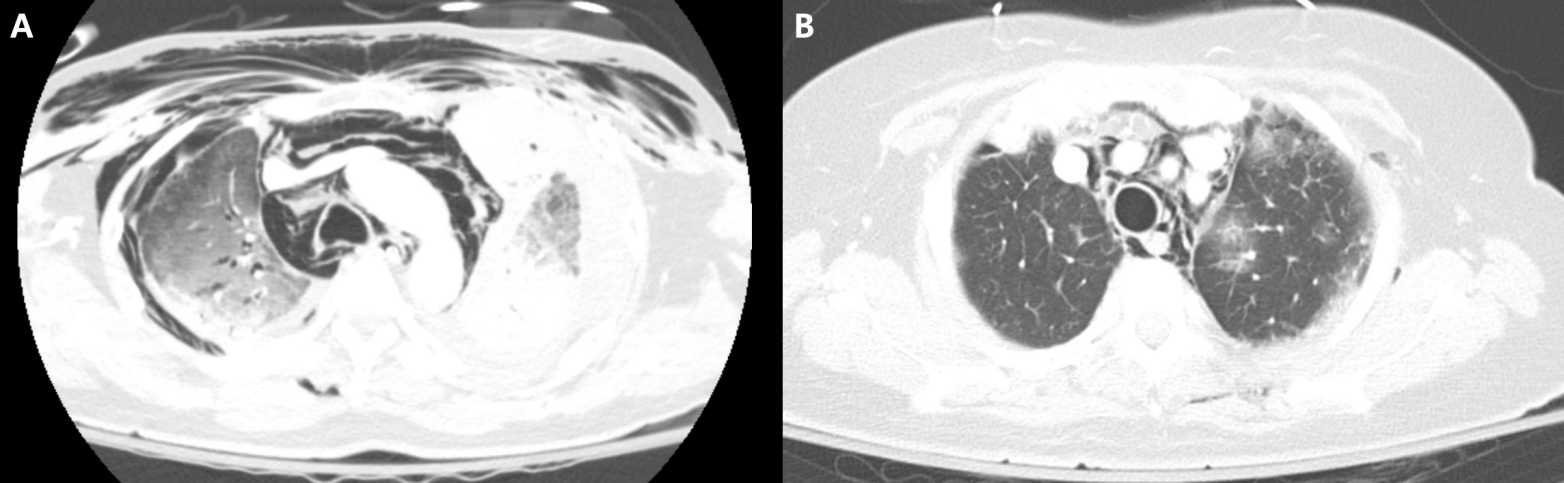
Chest CTs of pneumomediastinum found in patients with myositis-associated ILD. (A) shows a pneumomediastinum found in a patient with dermatomyositis-associated ILD (47, M). (B) also is a pneumomediastinum developed in a patient with dermatomyositis-associated ILD (62, F).

Spontaneous pneumomediastinum was observed as the initial manifestation of myositis-associated ILD in two patients (16.7%) while in the remaining 10 patients, pneumomediastinum developed at a median of 2 months after the diagnosis (interquartile range [IQR] 0.7–2.9 months) of ILD. Fig 1 illustrates the representative CT images of patients with pneumomediastinum from our study.

### Factors affecting mortality and survival analysis

During the follow-up period, 21 (30.0%) patients died. Table 2 presents the findings of the Cox proportional hazard analysis for the risk factors associated with mortality. Univariate Cox proportional hazard analysis revealed the development of pneumomediastinum (hazard ratio [HR] = 3.087, 95% CI 1.214–7.848, *p* = 0.018) and elevated levels of KL-6 (>1000.0 U/mL, HR = 4.110, 95% CI 1.210–13.963, *p* = 0.023) were statistically significant risk factors for mortality (Table 2). Following adjustment for the effect of smoking history, which exhibited a relative association with mortality but had no missing values, pneumomediastinum remained an independent and significant risk factor for mortality (HR = 2.829, 95% CI 1.100–7.270, *p* = 0.032) in the multivariate analysis.

**Table 2:** Cox proportional hazard analysis of risk factors for mortality in patients with myositis-associated ILD.

Parameters	Hazard ratio	95% confidence interval	*p*-value
Univariate analysis			
Age, years	1.014	0.979–1.052	0.432
Ever-smoker	0.486	0.161–1.464	0.199
Male sex	1.538	0.634–3.730	0.341
Malignancy	1.341	0.392–4.591	0.640
Pneumomediastinum	3.087	1.214–7.848	0.018
Baseline PFT			
FVC, % predicted	0.987	0.955–1.021	0.459
FEV1, % predicted	0.994	0.957–1.033	0.764
DLCO, % predicted	0.975	0.936–1.015	0.223
TLC, % predicted	0.973	0.931–1.017	0.219
6-minute walk test			
Meter (m)	0.996	0.990–1.002	0.163
Lowest O2 ≤ 88%	3.296	0.821–13.225	0.092
Initial laboratory data			
Aldolase, IU/L	0.989	0.952–1.027	0.568
LD, U/L	1.001	0.999–1.002	0.470
KL-6 > 1000.0 U/mL	4.110	1.210–13.963	0.023
Multivariate analysis^†^			
Ever-smoker	0.562	0.183–1.720	0.312
Pneumomediastinum	2.829	1.100–7.270	0.031

† Smoking history and pneumomediastinum were used as covariates in the multivariate analysis. KL-6 was excluded in the multivariate model despite its significance shown in the univariate analysis because of missing data (14/70). Desaturation was also excluded in the multivariate analysis model despite its relative significance in univariate analysis owing to missing data (20/70).

**Fig 2:**
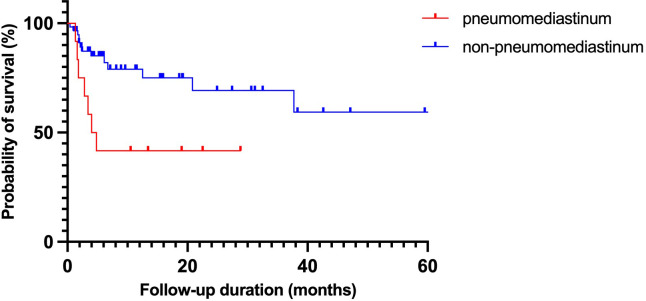
Kaplan–Meier curves of pneumomediastinum group and non-pneumomediastinum group.

Fig 2 shows the Kaplan–Meier curves divided into two groups based on whether patients had or did not have pneumomediastinum. The median survival was significantly shorter in the pneumomediastinum group than in the non-pneumomediastinum group (13.6 ± 3.7 vs. 77.7 ± 11.7 months, *p* = 0.013).

### Treatment and clinical outcomes

Corticosteroid therapy was initiated in all 70 patients, with 27.1% (19/70) receiving steroid pulse therapy. Additionally, 50 (71.4%) patients required additional immunosuppressive therapy, including mycophenolate mofetil (MMF), azathioprine, cyclosporin, and rituximab, listed in descending order of frequency in Table 3.

**Table 3:** Comparison of treatments between the pneumomediastinum and non-pneumomediastinum groups.

	Total (*n* = 70)	Pneumomediastinum (*n* = 12)	Non-Pneumomediastinum (*n* = 58)	*p-*value
Initial dose of corticosteroid in PD, mg	53.8 ± 75.6	45.5 ± 21.2	55.5 ± 82.6	0.680
Steroid pulse therapy^†^	19 (27.1%)	6 (50.0%)	13 (22.4%)	0.074
Other immunosuppressants	50 (71.4%)	10 (83.3%)	40 (69.0%)	0.487
MMF	29 (41.4%)	9 (75.0%)	20 (34.5%)	0.021
Azathioprine	17 (24.3%)	2 (16.7%)	15 (25.9%)	0.717
Cyclosporin A	15 (21.4%)	0 (0.0%)	15 (25.9%)	0.057
Rituximab	8 (11.4%)	3 (25.0%)	5 (8.6%)	0.132

PD = prednisolone, MMF = mycophenolate mofetil.

† In steroid pulse therapy, 500 mg of methylprednisolone was applied per day, four times daily.

A comparison between the pneumomediastinum and non-pneumomediastinum groups revealed a higher proportion of MMF usage in the pneumomediastinum group (9/12 (75.0%) vs. 20/58 (34.5%), *p* = 0.021) was spotted. Although the initial dosage of corticosteroid did not differ significantly differ between the two groups, there was a trend towards more frequent usage of steroid pulse therapy in the pneumomediastinum group (6/12 (50.0%) vs. 13/58 (22.4%), *p* = 0.074).

## Discussion

In our study, spontaneous pneumomediastinum emerged in 17.1% of patients and was identified as an independent risk factor for mortality (HR 2.829; CI 1.100–7.270; *p* = 0.031). Notably, pneumomediastinum primarily manifested in patients with dermatomyositis, with no cases associated with polymyositis. Moreover, individuals with spontaneous pneumomediastinum exhibited a significantly lower survival rate compared with those without pneumomediastinum.

ILD is the most prevalent extramuscular manifestation of IIM, occurring in 19–42% of all patients with IIM and consistently associated with poorer outcomes [14]. Particularly concerning are cases of rapidly progressive ILD (RP-ILD) in patients with IIM, which demand heightened vigilance due to reported mortality rates of up to 70–90% [15]. In our study, 21 (30.0%) cases resulted in death or necessitated lung transplantation during the median follow-up period of 5.7 (IQR 2.4–19.0). Previous studies conducted at our institution, albeit involving different patient cohorts, revealed that most patients with dermatomyositis- or polymyositis-associated ILD followed a chronic disease course. However, some cases exhibited an exceedingly acute and rapid progression; in fact, 72.7% expired within 1–2 months of diagnosis despite receiving intensive treatment [16]. Recent studies focusing on anti-melanoma differentiation-associated gene 5 (MDA-5) antibody-positive dermatomyositis have underscored the urgent need for aggressive therapeutic approaches, including steroid pulse therapy and early initiation of immunosuppressive therapy [17–19]. The identification of clinical parameters predictive of poor prognosis among patients with myositis-associated ILD is needed, other than autoantibodies.

Pneumomediastinum, characterized by the presence of extraluminal gas in the mediastinum, can result from various causes including alveolar rupture, tracheobronchial injury, or esophageal perforation [20]. Among these potential triggers, alveolar rupture induced by elevated trans-alveolar pressure or disruption of the mucosal barrier is recognized as a key underlying pathophysiological mechanism of pneumomediastinum [8]. In particular, bronchial mucosal disruption stemming from vasculopathy is believed to play a crucial role in the development of pneumomediastinum. This hypothesis finds support in the observed pathologic differences between subtypes of IIM; notably, specimens from patients with dermatomyositis exhibit a significantly higher burden of perivascular regional infiltration of B/T cells, which serves as a histologic indicator of vasculitis, compared with those from patients with polymyositis [21,22]. Previous studies have consistently reported a higher incidence of pneumomediastinum in dermatomyositis compared with polymyositis [7,8], a trend that aligns with the findings of our study.

Spontaneous pneumomediastinum can complicate various subtypes of ILD [23–25], although its incidence is relatively low. Pneumomediastinum might be observed in clinical course of IPF and is associated with poor outcomes [26,27]. However, the impact of spontaneous pneumomediastinum on mortality in other ILD types, including CTD-related and myositis-associated ILD, remains contentious. Giacomi et al [28] analyzed 25 cases of spontaneous pneumomediastinum in individuals with CTD-related ILD, reporting that 22 (88%) patients presented with short and benign courses without fatalities. Similarly, Okamoto et al. [29] conducted a retrospective review of 61 patients with CTD-related ILD, observing that clinically amyopathic dermatomyositis (CADM) was significantly more prevalent in the pneumomediastinum group compared with the non-pneumomediastinum cases (4/13 (31%) vs. 3/49 (6%), *p* = 0.03), although there was no difference in survival between the two groups. Likewise, Yoshida et al. [30] documented a survival case of pneumomediastinum complicated with dermatomyositis-associated ILD, suggesting that mortality primarily results from respiratory failure due to ILD itself, with pneumomediastinum alone not necessarily indicative of a poor prognosis. In contrast, a recent retrospective study conducted in Japan involving 119 patients with dermatomyositis/polymyositis-associated ILD revealed a significantly higher mortality rate in patients with spontaneous pneumomediastinum, particularly those with positive MDA-5 antibody [10], consistent with the findings of our study. Further investigations, including considerations of ethnic diversity or association with severity of ILD, are warranted to better understand the complex relationship between spontaneous pneumomediastinum and mortality in various ILD subtypes.

Recent recommendations have proposed various treatment strategies for myositis-associated ILD [31–33], although a definitive gold standard remains elusive. The cornerstone of treatment typically involves corticosteroid therapy in combination with immunosuppressants, with additional consideration given to intravenous immunoglobulin (IVIG), antifibrotics, and lung transplantation in refractory cases [14,34]. In our current study, the utilization of MMF was significantly higher (45.4% vs. 75.0%, *p* = 0.021), and there was a marginally significant increase in the proportion of patients receiving steroid pulse therapy (27.1% vs. 50.0%, *p* = 0.074), in the pneumomediastinum group compared with the non-pneumomediastinum group. This likely reflects the heightened severity of ILD in patients with pneumomediastinum. The role of corticosteroid therapy in pneumomediastinum remains controversial. While a previous study demonstrated that steroid therapy improved survival rates in dermatomyositis-associated ILD complicated with spontaneous pneumomediastinum [35], corticosteroid use has also been implicated as a potential risk factor for the development of spontaneous pneumomediastinum in CTD-related ILD [36]. Further investigations are needed to elucidate the relationship between steroid therapy and its optimal dosage in the management of pneumomediastinum complicated with dermatomyositis-associated ILD.

There are several limitations to our study. First, due to its retrospective nature and being conducted at a single center, there were instances of missing data, particularly regarding PFT, 6MWT, and laboratory results. Second, the absence of pneumomediastinum cases in patients with polymyositis precluded the analysis of whether the subtype of IIM was a significant risk factor for the development of pneumomediastinum. Third, we did not investigate different myositis-associated antibodies (MAAs), including MDA-5. The utilization of myositis panels is relatively new in South Korea, and its limited availability under national healthcare insurance coverage restricts its clinical use. However, recent evidence suggests that MDA-5-positive dermatomyositis associated with ILD often exhibits rapid progression with high mortality rates, with the possibility of increased incidence of spontaneous pneumomediastinum [10,15]. Given the significant impact of MAAs on patient outcomes, further investigations are warranted to assess their role in the development and prognosis of pneumomediastinum, which could be incorporated into future study designs. Finally, even after thoroughly reviewing all medical records, we were unable to accurately determine the scores required for EULAR/ACR classification from the biopsy specimens. Moreover, detailed descriptions of muscle weakness severity and precise documentation of skin lesions were unavailable for most patients, making accurate scoring impossible in the majority of cases. Although all patient cases were confirmed by rheumatologists, some ‘possible’ or ‘probable’ IIM cases might have been included. However, given the thorough medical record review, confirmation by rheumatologists, and registration in South Korea’s Rare and Intractable Disease (RID) program, it is highly unlikely that the enrolled patients did not meet the diagnostic criteria for IIM.

## Conclusion

In conclusion, our findings suggest that pneumomediastinum could serve as a significant prognostic indicator in patients with myositis-associated ILD. Physicians should be cognizant of the heightened risk associated with pneumomediastinum, particularly in the dermatomyositis-associated ILD.

## Supporting information

S1 Data (XLSX)

## Abbreviations

ASS: Anti-synthetase syndrome
CADM: Clinically amyopathic dermatomyositis
CT: Computed tomography
CTD: Connective tissue disease
DLco: Diffusing capacity of the lung for carbon monoxide
DM: Dermatomyositis
EULAR/ACR: European League Against Rheumatism/American College of Rheumatology
FVC: Forced vital capacity
HR: Hazard ratio
IBM: Inclusion body myositis
IIM: Idiopathic inflammatory myositis
ILD: Interstitial lung disease
IQR: Interquartile range
IRB: Institutional Review Board
IVIG: Intravenous immunoglobulin
KL-6: Krebs von den Lungen-6
LD: Lactose dehydrogenase
MAA: Myositis-associated antibodies
MDA-5: Melanoma differentiation-associated protein 5
MMF: Mycophenolate mofetil
PD: Prednisolone
PFT: Pulmonary function test
PM: Polymyositis
RP-ILD: Rapidly progressing interstitial lung disease
TLC: Total lung capacity
6MWT: 6-minute walk test.
